# Resolution of Inflammation: Leukocytes and Molecular Pathways as Potential Therapeutic Targets

**DOI:** 10.3389/fimmu.2013.00256

**Published:** 2013-08-27

**Authors:** János G. Filep

**Affiliations:** ^1^Research Center, Maisonneuve-Rosemont Hospital, University of MontrealMontreal, QC, Canada

**Keywords:** inflammation, leukocytes, molecular pathway, therapeutic target, inflammatory response

Inflammation is the principal defense mechanism against invading pathogens and host injury. Neutralization and elimination of the offending insult ideally prompts resolution of inflammation and restoration of tissue homeostasis. Excessive or dysregulated inflammatory responses together with impaired repair processes results in non-resolving inflammation and persisting tissue damage, which is now considered as a critical component of many chronic human diseases. Ground-breaking work during the past decade has led to the recognition that resolution of inflammation is a tightly controlled active process that involves generation of a new class of lipid mediators, proteins, and autacoids. These endogenous molecules possess overlapping but not fully identical biological properties and may act simultaneously or sequentially to regulate processes that are essential for timely and complete resolution of inflammation. Among these mechanisms are sequestering and suppression of generation of pro-inflammatory cytokines, inhibition of inflammatory cell trafficking into inflamed sites, promotion of neutrophil apoptosis, and macrophage polarization into M2 (alternatively activated or pro-resolution) phenotype, and facilitation of removal of apoptotic neutrophils and other cell types via efferocytosis and tissue repair.

During the past years much progress has been made in the identification and characterization of pro-resolution mediators and signaling circuits. Of these, protein mediators, such as annexin A1, and novel classes of lipid mediators, including arachidonic acid-derived lipoxins, and omega-3 polyunsaturated fatty acid-derived resolvins, protectins, and maresins exhibit dual anti-inflammatory and pro-resolution properties. Extensive research has characterized biosynthetic pathways, G-protein-coupled receptors, downstream intracellular signaling pathways, and identified several microRNAs that are involved in the regulation of pro-resolving molecular and cellular circuits. While in inflamed sites neutrophils interact with other cells in their immediate vicinity to produce these lipid mediators, eosinophil granulocytes can also generate pro-resolving mediators from omega-3 polyunsaturated fatty acids, indicating an important role for this type of leukocytes in inflammatory resolution. Resolvins facilitate macrophage polarization into the M2 phenotype. Lipoxins and resolvins interfere with β2 integrin-mediated outside in signaling that in addition to governing leukocyte trafficking also modulates neutrophil apoptosis, another control point in resolving inflammation. Data emerging from a variety of experimental models demonstrated that resolvins and protectins limit adaptive immunity in asthma, attenuate adipose tissue inflammation and metabolic dysfunctions, including type-2 diabetes mellitus-associated insulin resistance and non-alcoholic fatty acid liver disease, and protect the kidney against diabetic nephropathy. The glucocorticoid-regulated protein annexin A1 may act in concert with lipoxins to limit inflammation underlying myocardial ischemia/reperfusion, arthritis, multiple sclerosis, and sepsis. Altering local adenosine signaling affects leukocyte activation and trafficking, and may restore tissue homeostasis in liver injury and rheumatoid arthritis. The nuclear proteins proliferating cell nuclear antigen (PCNA) and myeloid nuclear differentiation antigen (MNDA) play opposing roles in modulating neutrophil apoptosis in patient with sepsis. Targeting the complement C5a receptors C5Ra and C5L2 may attenuate excessive inflammation in sepsis and autoimmune diseases. Intracellular molecular mechanisms have also been implicated in dampening inflammation. The NOD-like receptors NOD1 and NOD2 recognize intracellular pathogens and activate transcriptional responses that would lead to restriction of infection. Suppression of IFNγ production in T lymphocytes by allopurinol may attenuate cell-mediated inflammatory diseases.

This Research Topic focuses on articles that discuss the molecular mechanisms governing the function and fate of leukocytes during inflammation and identify potential targets for development of innovative therapeutic approaches for the treatment of inflammatory pathologies based on mimicking and/or activating endogenous pro-resolution mechanisms.

